# Electro-magnetic analysis of high-frequency digital signal processors

**DOI:** 10.1186/s40064-016-2999-2

**Published:** 2016-08-09

**Authors:** Bing Li, Mingzhu Lei, Meiyuan Chen, Lanyong Zhang

**Affiliations:** College of Automation, Harbin Engineering University, Heilongjiang, China

**Keywords:** High-frequency digital processors, Electro-magnetic radiation, Dipole, Prediction analysis

## Abstract

High-frequency digital signal processors are increasingly suffering from electro-magnetic interference, due to its ever-increasing integration level and operation speed. The accurate prediction of its electro-magnetic effects require less effort to be spared in the design procedures to obtain better electro-magnetic compatibility and to avoid later modifications that are lengthy and expensive. In this paper, the dipole method is implemented to predict the magnetic impacts of DSP6713 system in order to reduce its design costs.

## Background

Control systems based on high-frequency digital signal processors are playing a more and more important roles in control, signal processing, and telecommunications and others (Hamza et al. 2013; Kaczmarek et al. 2010). However, along with the ever-increasing operating frequency, the electrical equipment and control boards are suffering from greater electro-magnetic interference (EMI) than ever before (Devabhaktuni et al. 2013; He et al. 2011; Wang et al. 2013). In this paper, the electromagnetic theory and its application to the electromagnetic compatibility are used to analyse the system electro-magnetic compatibility. As a result, the electro-magnetic compatibility is considered in the design stage to reduce the risk of necessary work in the later stages (Coenen et al. 2012).

At present, the universal analysis methods of electromagnetic radiation include mode expansion method, integral equation method, equivalent current method, equivalent magnetic current method, and equivalent dipole and its array. Dipole model is adopted to equivalent of PCB electromagnetic radiation in this paper. Under analyzing of the dipole radiation characteristics, equivalent model of dipole array can be derived in order to predict the electromagnetic radiation characteristics of high frequency digital signal processor. Finally, the results of simulations and the measured data are compared, which verifies the practicability of the equivalent dipole model.

## Electro-magnetic model based on dipole

### Dipole model

The time-variant current in the high-frequency digital signal processors has been considered as one of the main contributing factors to their EMI (Shin et al. 2010). This time-variant current, however, generally exists in one of the two possible forms: magnetic current elements in the magnetic field sources and electric dipoles in the electric field sources (Kim and Li 2010; Wu et al. 2013). Based on this description above, the electric dipole and magnetic dipole can be selected to replace the magnetic field and electric field of the time varying current in the integrated circuit.

#### Antenna radiation characteristics of magnetic current elements

**Fig. 1:**
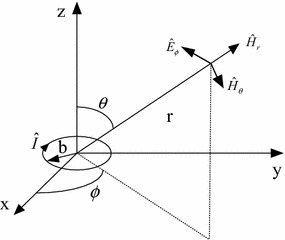
Magnetic dipoles

The time-variant current, if existing in the closed loop or small current loop of the magnetic field source, is defined as magnetic current elements or magnetic dipole. As is depicted in Fig. 1, a ring with the radius of *b* locating on the surface *xy* carries a current vector $\hat{I}$. Traditionally, when describing antennas, the position of a specific point is described by its radial distance *r* measured from the origin, angle *θ* between the radial line and axis *z*, and angle $\phi$ between its projection on surface *xy* and axis *x*. Assuming a negligible perimeter (i.e. 2*πb* < λ_0_/10), the resulting magnetic dipole moment can be expressed as:

1 $$ \hat{m} = \hat{I}\pi b^{2} $$

where *πb*^2^ stands for area surrounded by the ring. Hence, the radiation filed can be denoted as:

2 $$ \hat{E}_{r} = 0 $$

3 $$ \hat{E}_{\theta } = 0 $$

4 $$ \hat{E}_{\phi } = - j\frac{{\omega \mu_{0} \hat{m}\beta_{0}^{2} }}{4\pi }\sin \theta \left( {j\frac{1}{{\beta_{0} r}} + \frac{1}{{\beta_{0}^{2} r^{2} }}} \right)e^{{ - j\beta_{0} r}} $$

and

5 $$ \hat{H}_{r} = 2j\frac{{\omega \mu_{0} \hat{m}\beta_{0}^{2} }}{{4\pi \eta_{0} }}\cos \left( {\frac{1}{{\beta_{0}^{2} r^{2} }} - j\frac{1}{{\beta_{0}^{3} r^{3} }}} \right)e^{{ - j\beta_{0} r}} $$

6 $$ \hat{H}_{\theta } = j\frac{{\omega \mu_{0} \hat{m}\beta_{0}^{2} }}{{4\pi \eta_{0} }}\sin {\kern 1pt} {\kern 1pt} \theta \left( {j\frac{1}{{\beta_{0} r}} + \frac{1}{{\beta_{0}^{2} r^{2} }} - j\frac{1}{{\beta_{0}^{3} r^{3} }}} \right)e^{{ - j\beta_{0} r}} $$

7 $$ \hat{H}_{\phi } = 0 $$

where $\eta_{0} = \sqrt {\mu_{0} /\varepsilon_{0} }$ stands for the intrinsic impedance of free space, *ε*_0_ for its dielectric constant, and *μ*_0_ for its permeability respectively ($\varepsilon_{0} = 1/36\pi \times 10^{ - 9} ,\,\mu_{0} = 4\pi \times 10^{ - 7}$).

For magnetic dipoles in close-in region (*kr* ≪ 1 or *r* < *λ*/2*π*) and *k* stands for wave number, near field is expected, where magnetic field *H* is inversely proportional to distance *r*^3^ (*H* ∝ 1/*r*^3^), and electric field *E* is inversely proportional to distance *r*^2^, and the wave impedance *Z* = *jωμ*_0_*r*. Therefore, the near field generated by magnetic dipoles is of low impedance since it is mainly magnetic field.

For the far field (*kr* ≫ 1 or *r* > *λ*/2*π*), the amplitude ratio of electric field and magnetic field is wave impedance (*E*/*H* = *Z*).

#### Antenna radiation characteristics of electric dipoles (current element)

**Fig. 2:**
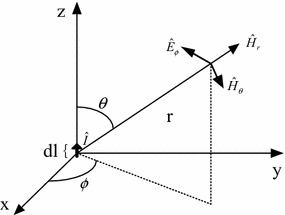
Electric dipoles

Electric field source of electric dipoles is commonly defined as current element. Electric dipoles consist of infinitesimal current vector $\hat{I}$ with the length of *dl*, assuming current elements share the same phase and amplitude at all points, as shown in Fig. 2. Spherical coordinate can also be used to describe the electro-magnetic field generated by electric dipoles, like that generated by magnetic dipoles. Magnetic intensity vector can be written as:

8 $$ \hat{H}_{r} = 0 $$

9 $$ \hat{H}_{\theta } = 0 $$

10 $$ \hat{H}_{\phi } = \frac{{\hat{I}dl}}{4\pi }\beta^{2} \sin \theta \left( {j\frac{1}{{\beta_{0} r}} + \frac{1}{{\beta_{0}^{2} r^{2} }}} \right)e^{{ - j\beta_{0} r}} $$

Electric field intensity vector can be described as:

11 $$ \hat{E}_{r} = 2\frac{{\hat{I}dl}}{4\pi }\eta_{0} \beta_{0}^{2} \cos \theta \left( {\frac{1}{{\beta_{0}^{2} r^{2} }} - j\frac{1}{{\beta_{0}^{3} r^{3} }}} \right)e^{{ - j\beta_{0} r}} $$

12 $$ \hat{E}_{\theta } = \frac{{\hat{I}dl}}{4\pi }\eta_{0} \beta_{0}^{2} \sin \theta \left( {j\frac{1}{{\beta_{0} r}} + \frac{1}{{\beta_{0}^{2} r^{2} }} - j\frac{1}{{\beta_{0}^{3} r^{3} }}} \right)e^{{ - j\beta_{0} r}} $$

13 $$ \hat{E}_{\phi } = 0 $$

Similarly, for close-in region (*kr* ≪ 1 or *r* < *λ*/2*π*), electric field *E* is inversely proportional to distance *r*^3^ (*E* ∝ 1/*r*^3^), and magnetic field *H* is inversely proportional to distance *r*^2^, and the wave impedance *Z* = (*jω*_0_*r*)^−1^. Therefore, the near field generated by electric dipoles is of high impedance since it is mainly electric field.

For the far field (*Kr* ≫ 1 or *r* > *λ*/2*π*), the amplitude ratio of electric field and magnetic field is wave impedance (*E*/*H* = *Z*).

### Equivalent dipole model of integrated circuit

**Fig. 3:**
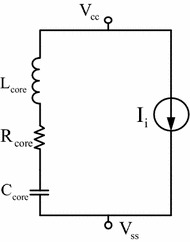
Equivalent circuit model

In this paper, the integrated circuit emission model (ICEM), which is widely accepted by IC manufacturers and designers to analyse, optimize and predict parameters, is used for electro-magnetic compatibility modelling and analysis. In order to reduce the complexity of digital IC, the ICEM model is implemented here for modelling purposes, since the interferences are mainly radiation and conduction based. The distribution network model in the ICEM model is derived with simplified equivalence and impedance is represented by a series circuit of *R*, *L* and C. Hence, simplified equivalence, and the impedance is depicted in Fig. 3.

**Fig. 4:**
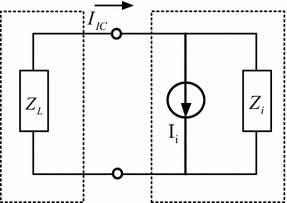
Sketch of measurement instruments

**Fig. 5:**
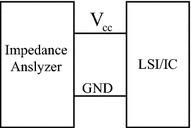
Measuring *Z*_*i*_ with an impedance analyser

As is shown in Fig. 4, in order to determine parameters of the circuit, *Z*_*L*_ stands for the external impedance of the supply, *I*_*i*_ for the equivalent internal current source of the LSI/IC under measurement, and *Z*_*i*_ for the equivalent internal impedance of the device under measured. The device internal impedance can be measured by an impedance analyser, as is demonstrated in Fig. 5. The resonance frequency *f*_*r*_ can then be observed from the impedance diagram. The resulting parameters (*R*, *L* and *C*) are finally calculated based on Eqs. (14) and (15).

14 $$ f_{r} = \frac{1}{{2\pi \sqrt {LC} }} $$

15 $$ Z_{i} = \left\{ {\begin{array}{*{20}l} {1/2\pi fC}  &\quad {f < f_{r} } \hfill \\ R \hfill &\quad {f = f_{r} } \hfill \\ {2\pi fL} \hfill &\quad {f > f_{r} } \hfill \\ \end{array} } \right. $$

**Fig. 6:**
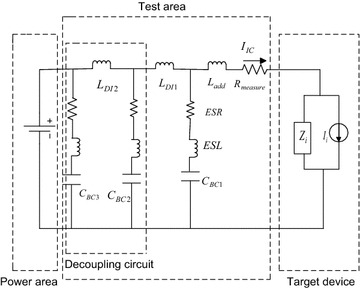
Measuring *I*_*IC*_ with a spectrum analyser

The external circuit current *I*_*IC*_ can be measured by a spectrum analyser and the respective sketch is shown in Fig. 6.

Therefore, the internal current source *I*_*i*_ can be derived based on Eq. (16):

16 $$ I_{i} = \frac{{Z_{i} + Z_{L} }}{{Z_{i} }}I_{IC} $$

### DDC model of PCB

**Fig. 7:**
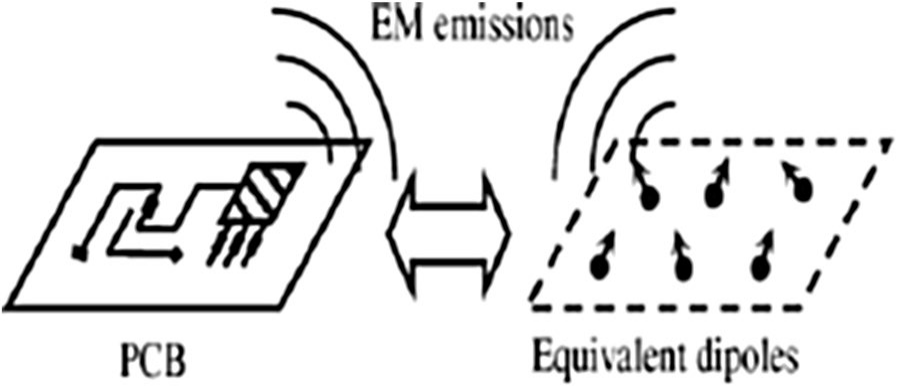
The basic principle of the equivalent dipole method

DDC model is the equivalent dipole model derived based on near field scanning, which essentially replaces PCB radiation with a series of infinitesimal dipoles. Generally speaking, the establishment of the model does not require much technical knowledge of PCB layouts, and, hence, only simple geometric sizes are necessary. Since most PCBs are relatively thin, its model can be divided and described as matrix arrays, which are generated by dipoles at arbitrary locations on the PCB surface, as is shown in Fig. 7.

Many dipoles are distributed in a single large integrated circuit in various directions. In order to simplify the problems under investigation, each dipole is dissolved into three components *M*^*x*^, *M*^*y*^ and *M*^*z*^. Based on equivalence principles, the dipole model should yield the same electro-magnetic field as the PCB. Therefore, the moment of the dipole (magnitude and phase) and direction are determined by the tangential component of the electro-magnetic field located on close-in plate. In Cartesian coordinate system, the radiation at (*x*, *y*, *z*) generated by infinitesimal dipoles *M*^*z*^, can be expressed as

17 $$ \left\{ \begin{aligned} H_{x} & = M^{z} \frac{{jke^{ - jkr} }}{{4\pi r^{4} }}(x - x_{0} )(z - z_{0} )\left( {jkr + 3 + \frac{3}{jkr}} \right) \\ & = M^{z} \xi_{x}^{z} \\ H_{y} & = M^{z} \frac{{jke^{ - jkr} }}{{4\pi r^{4} }}(y - y_{0} )(z - z_{0} )\left( {jkr + 3 + \frac{3}{jkr}} \right) \\ & = M^{z} \xi_{y}^{z} \\ H_{z} & = M^{z} \frac{{jk^{2} e^{ - jkr} }}{4\pi r}\left[ {\frac{{(z - z_{0} )^{2} }}{{r^{2} }}\left( {j + \frac{3}{kr} + \frac{3}{{jk^{2} r^{2} }}} \right) - \left( {j + \frac{1}{kr} + \frac{1}{{jk^{2} r^{2} }}} \right)} \right] \\ & = M^{z} \xi_{z}^{z} \\ \end{aligned} \right. $$

Similarly, the radiations at (*x*, *y*, *z*) by dipole *M*^*y*^ and *M*^*x*^ can be written as:

18 $$ \left\{ \begin{aligned} H_{x} & = M^{y} \frac{{jke^{ - jkr} }}{{4\pi r^{4} }}(x - x_{0} )(y - y_{0} )\left( {jkr + 3 + \frac{3}{jkr}} \right) \\ & = M^{y} \xi_{x}^{y} \\ H_{y} & = M^{y} \frac{{jk^{2} e^{ - jkr} }}{4\pi r}\left[ {\frac{{(y - y_{0} )^{2} }}{{r^{2} }}\left( {j + \frac{3}{kr} + \frac{3}{{jk^{2} r^{2} }}} \right) - \left( {j + \frac{1}{kr} + \frac{1}{{jk^{2} r^{2} }}} \right)} \right] \\ & = M^{y} \xi_{y}^{y} \\ H_{z} & = M^{y} \frac{{jke^{ - jkr} }}{{4\pi r^{4} }}(y - y_{0} )(z - z_{0} )\left( {jkr + 3 + \frac{3}{jkr}} \right) \\ & = M^{y} \xi_{z}^{y} \\ \end{aligned} \right. $$

19 $$ \left\{ \begin{aligned} H_{x} & = M^{x} \frac{{jk^{2} e^{ - jkr} }}{4\pi r}\left[ {\frac{{(x - x_{0} )^{2} }}{{r^{2} }}\left( {j + \frac{3}{kr} + \frac{3}{{jk^{2} r^{2} }}} \right) - \left( {j + \frac{1}{kr} + \frac{1}{{jk^{2} r^{2} }}} \right)} \right] \\ & = M^{x} \xi_{x}^{x} \\ H_{y} & = M^{x} \frac{{jke^{ - jkr} }}{{4\pi r^{4} }}(y - y_{0} )(x - x_{0} )\left( {jkr + 3 + \frac{3}{jkr}} \right) \\ & = M^{x} \xi_{y}^{x} \\ H_{z} & = M^{x} \frac{{jke^{ - jkr} }}{{4\pi r^{4} }}(x - x_{0} )(z - z_{0} )\left( {jkr + 3 + \frac{3}{jkr}} \right) \\ & = M^{x} \xi_{z}^{x} \\ \end{aligned} \right. $$

where *k* stands for wave number and *r* represents the distance between the dipole and the measured point:

20 $$ r = \sqrt {(x - x_{0} )^{2} + (y - y_{0} )^{2} + (z - z_{0} )^{2} } $$

The magnitude and phase of the tangential component of the close-in magnetic field along the directions of *H*_*x*_ and *H*_*y*_ can be derived by *p* × *q* discrete sampling points. Two adjustable probes are used during the measurement. For fixed reference probe, the measuring probe moves on the sweeping plate, and the obtained signals are delivered to vector network analyser operating with external supply, which is used to record the ratios of two signals with complex formation to describe the magnitude and direction of the field. In fact, the field of each testing point is the combination of the effects of all the equivalent dipoles. On the condition that there are *m* sampling points and *n* dipoles available, and the tangential components of the magnetic field at the measured discrete points can be represented by a two dimensional matrix, the following linear matrix can be used to describe the its relationship with dipole source magnetic field vector:

21 $$ \left[ {\begin{array}{*{20}l} {\xi_{x}^{x} } \hfill &\quad {\xi_{x}^{y} } \hfill &\quad {\xi_{x}^{z} } \hfill \\ \end{array} } \right]_{m \times n} \left[ {\begin{array}{*{20}l} {M^{x} } \hfill \\ {M^{y} } \hfill \\ {M^{z} } \hfill \\ \end{array} } \right]_{n \times 1} = \left[ {H_{x} } \right]_{m \times 1} $$

22 $$ \left[ {\begin{array}{*{20}l} {\xi_{y}^{x} } \hfill &\quad {\xi_{y}^{y} } \hfill &\quad {\xi_{y}^{z} } \hfill \\ \end{array} } \right]_{m \times n} \left[ {\begin{array}{*{20}l} {M^{x} } \hfill \\ {M^{y} } \hfill \\ {M^{z} } \hfill \\ \end{array} } \right]_{n \times 1} = \left[ {H_{y} } \right]_{m \times 1} $$

where the superscripts represent the dipole components while the subscripts represent the measured field components.

Since the coefficients *ξ*_*x*_ and *ξ*_*y*_ are related to the position and frequency, the time of each dipole can be solved by solving the reverse problem of linear equations in the frequency domain. In order to derive the unique solution of *M*, the number of dipoles should be no more than the measured points (*n* ≤ *m*). With the increasing accuracy of the near field scan, both the close-in and far magnetic field characteristics of PCB can be fully represented by its equivalent dipole source. For the coefficient *ξin* the above equation is merely related with the position and frequency, the equivalent dipoles can be obtained by solving the above equations.

## Electro-magnetic interference modeling of DSP6713 board

**Fig. 8:**
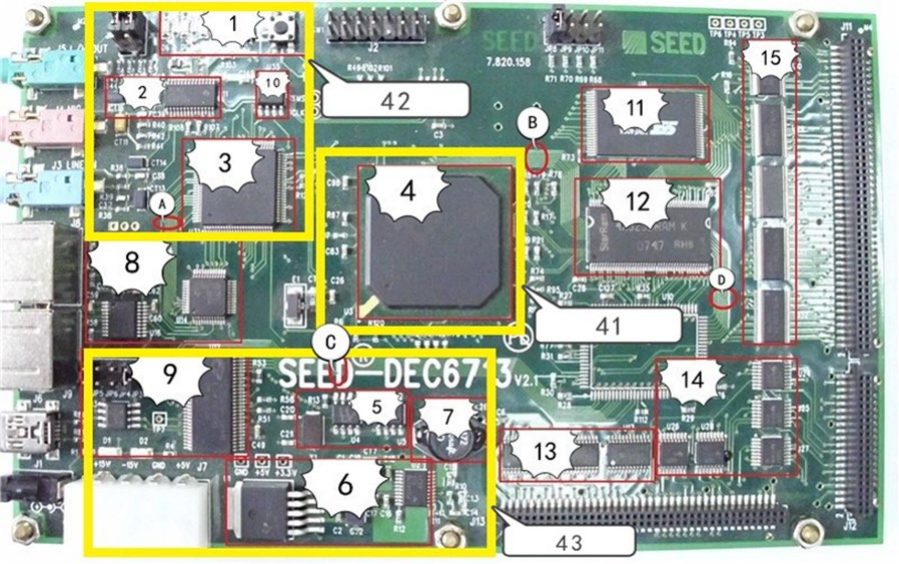
EMC testing diagram of DSP6713 board

DSP6713 board, depicted in Fig. 8, is developed based on TMS320C6713 DSP manufactured by TI, with a maximum operating speed of 1350 MIPS. In this figure, Region 1 stands for the reset chip, Region 2 for audio signal coder, Region 3 for logic control chip, Region 4 for DSP, Region 5 for clock distributor, Region 6 for standard voltage chip, Region 7 for power inductor, Region 8 for communication chip, Region 9 for USB receiving and sending controller, Region 10 for coder, Region 11 for memory, Region 12 for RAM, Region 13 for voltage level adaptor, and Region 15 for MUX.

In this section, the regional electro-magnetic interference model, interference at specific point by PCB, and PCB spectrum sweeping model are presented for DSP6713 board.

### Regional electro-magnetic interference model

Since the IC can be represented by the dipole model in “Equivalent dipole model of integrated circuit” section, it can further be reduced to be a ring with sufficiently small area, as shown in Fig. 1.

If $\hat{I}$ represents current vector and *b* represents the equivalent radius, the close-in magnetic field of the magnetic dipoles can be derived based on its theories as:

23 $$ \hat{H} \approx \hat{H}_{r} = \frac{{\omega \mu_{0} \hat{I}b^{2} \beta_{0}^{2} }}{{2\eta_{0} }}\cos {\kern 1pt} {\kern 1pt} {\kern 1pt} \theta \left( {j\frac{1}{{\beta_{0}^{2} r^{2} }} + \frac{1}{{\beta_{0}^{3} r^{3} }}} \right)e^{{ - j\beta_{0} r}} $$

where $\eta_{0} = \sqrt {\mu_{0} /\varepsilon_{0} }$ stands for intrinsic impedance of free space, *ε*_0_ for dielectric constant, *μ*_*0*_ for permeability ($\mu_{0} = 4\pi \times 10^{ - 7} ,\,\beta_{0} = \omega \sqrt {\mu_{0} \varepsilon_{0} }$), *r* for the distance to the center of IC equivalent model, and *θ* as the angle between the line and axis *z*.

### Interference at specific point by PCB

In this paper, based on electro-magnetic field measurement using the method outlined in “DDC model of PCB” section, the PCB can be represented by 9 equivalent dipoles, whose x, y, z components $$ M_{i}^{x} $$, $$ M_{i}^{y} $$, $$ M_{i}^{z} $$$$ \left( {1 \le i \le 9} \right) $$ can be calculated with the coefficients $$ \xi_{xi}^{x} $$, $$ \xi_{xi}^{y} $$,$$ \xi_{xi}^{z} $$,$$ \xi_{yi}^{x} $$,$$ \xi_{yi}^{y} $$,$$ \xi_{yi}^{z} $$, $$ \xi_{zi}^{x} $$,$$ \xi_{zi}^{y} $$, $$ \xi_{zi}^{z} $$, along the direction of x, y, z. Therefore, the electro-magnetic radiation at specific point by PCB can be expressed as:

24 $$ \left[ {\begin{array}{*{20}l} {\xi_{z}^{x} } \hfill &\quad {\xi_{z}^{y} {\kern 1pt} } \hfill &\quad {\xi_{z}^{z} } \hfill \\ \end{array} } \right]_{1 \times n} \left[ {\begin{array}{*{20}l} {M^{x} } \hfill \\ {M^{y} } \hfill \\ {M^{z} } \hfill \\ \end{array} } \right]_{n \times 1} = H_{z} \approx H $$

where *n* denotes the number of equivalent dipoles. The entire field can be approximated with a function of only a variable z, without x and y.

Note that the *H* obtained here are magnetic field intensity for specific frequency. However the signals are characterized by digital pulses, which are stochastic in nature, and the spectrum of such signals is expected to be very broad. Here, the equivalent dipole model has limitations, which predict multiple interferences only by using different equivalent dipole models respectively. So the whole spectrum (1–970 MHz) is divided into 50 frequency ranges linearly. And the magnetic field at the point can be obtained by 50 fields under respective frequency:

25 $$ \left[ {\begin{array}{*{20}l} {\xi_{z}^{x} } \hfill &\quad {\xi_{z}^{y} } \hfill &\quad {\xi_{z}^{z} } \hfill \\ \end{array} } \right]_{k \times n} \left[ {\begin{array}{*{20}l} {M^{x} } \hfill \\ {M^{y} } \hfill \\ {M^{z} } \hfill \\ \end{array} } \right]_{n \times 1} = \left[ {H_{z} } \right]_{k \times 1} $$

### EMI sweeping model of system board

The Dimensions of DSP6713 system board is 16 × 10 cm. The entire board can be replaced by 100 × 100 points in the model, and, as a result, the sweeping model can be represented by magnetic field of these points. Based on Eq. (24), the following equation can be used to approximate the electric magnetic field induced by these points at the point with a distance of 1 cm to the board:

26 $$ \begin{aligned} \left[ H \right]_{i \times j} & =  H_{z} (i,j) \\ & = \left[ {\begin{array}{*{20}l} {\xi_{z}^{x} (i,j)} \hfill &\quad {\xi_{z}^{y} (i,j)} \hfill &\quad {\xi_{z}^{z} (i,j)} \hfill \\ \end{array} } \right]_{1 \times n} \left[ {\begin{array}{*{20}l} {M^{x} } \hfill \\ {M^{y} } \hfill \\ {M^{z} } \hfill \\ \end{array} } \right]_{n \times 1}  \hfill \\ &\quad 1 \le i,j \le 100{\kern 1pt} \\ \end{aligned} $$

where *H*_*z*_(*i*, *j*) denotes the magnetic field intensity at the *i* × *j* point, while $\xi_{z}^{x} (i,j)$, $\xi_{z}^{y} (i,j){\kern 1pt}$ and $\xi_{z}^{z} (i,j)$ represent the coefficients along *x*, *y*, *z*.

## Electro-magnetic interference simulation and analysis of DSP6713 system board

### Electro-magnetic interference simulation and analysis of DSP6713 system board

**Fig. 9:**
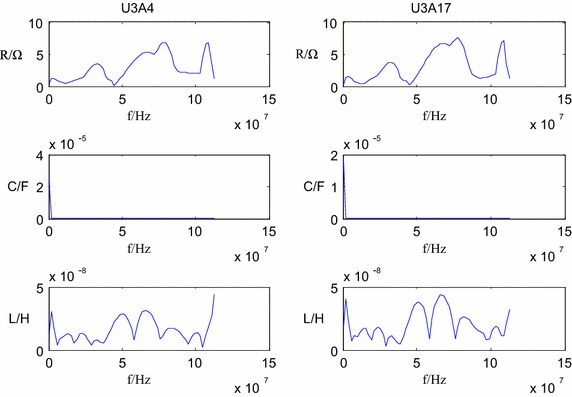
DSP parameters

The vector analyzer PNA3628 is used to measure various chip impedance of embedded equipment. Since the equivalent impedance model in Fig. 3 is formed by connecting equivalent resistor, capacitor and inductor in series, the parameters can be derived based on least square approximation. Due to space limit, the following is only used to demonstrate the respective model parameters of Region 4 of DSP. The parameters of DSP are shown in Fig. 9.

**Fig. 10:**
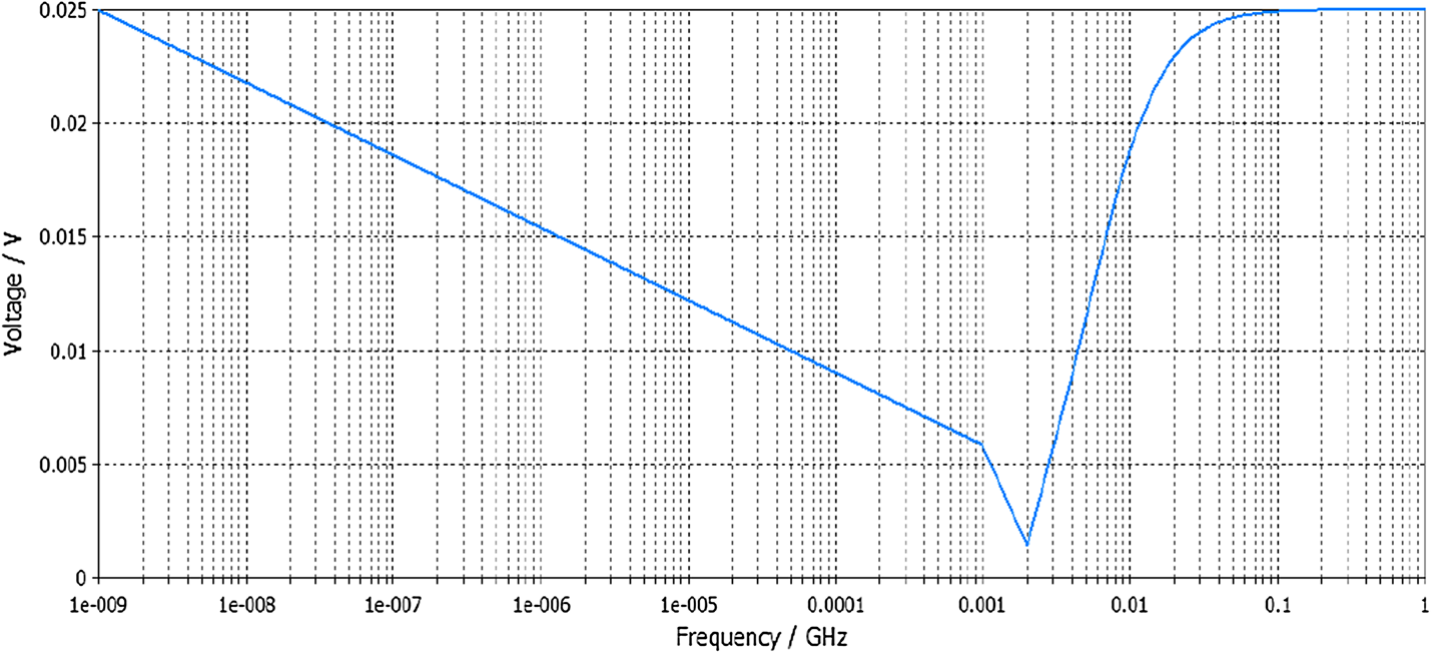
Frequency response of DSP

The resulted chip parameters and frequency response based on simulation are shown in Fig. 10.

**Fig. 11:**
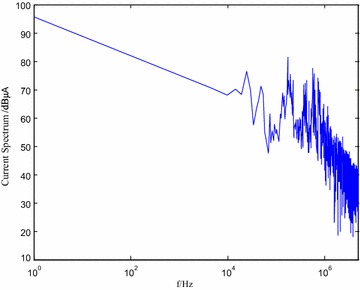
Current spectrum of DSP

The current spectrum based on Fourier transformation and the simulation results by equivalent dipole model are displayed in Fig. 11.

It can be observed from the figure that the maximum current is lower than 100 dB μA. Although the general trend of the current spectrum is decreasing, for frequency of MHz or higher, substantial current harmonics can be observed (even up to 60–80 dB μA for some chips), which severely affects electro-magnetic interference.

**Fig. 12:**
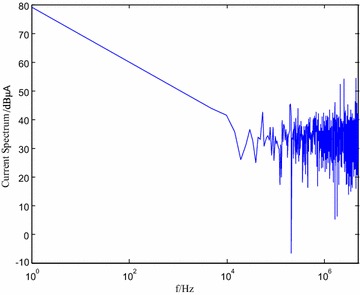
Current spectrum of DSP

The equivalent current spectrum is shown in Fig. 12 by performing Fourier transformation of the time domain signals by oscilloscope.

It can be observed from Fig. 12 that the maximum current is lower than 80 dB μA. Although the general trend of the current spectrum is decreasing, for frequency of MHz or higher, substantial current harmonics can be observed (even up to 30 dB μA-55 dB μA for some chips), which severely affects electro-magnetic interference, which is similar to the above current spectrum analysis and model based on equivalent dipole.

### Comparison of simulation and experiment results

**Fig. 13:**
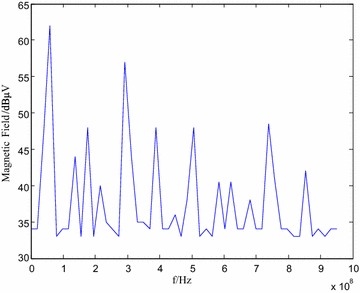
Simulation results of region 4 radiation

**Fig. 14:**
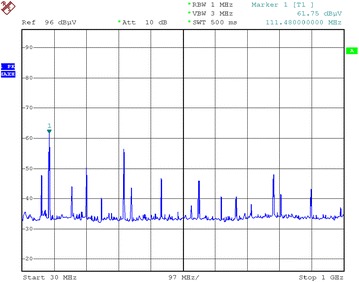
Experiment results of region 4 radiation

A result of the simulation for DSP Region 4 radiation is plotted in Fig. 13. DSP6713 system board is then subjected to the radiation test in a standard laboratory, with a sweeping spectrum of 30 MHz–1 GHz (1 MHz step), a reference voltage of 96 dB μV, and the measuring device attenuation of 10 dB. The experiment results are demonstrated in Fig. 14.

The comparison of simulation and experiment results of Region 4 radiation reveals that: (1) the general tendency is the same in the range from 30 MHz to 1 GHz since the radiation level decays with increasing frequency; (2) the radiation reaches its maximum of 63 dB μA at 80 MHz. The radiation level in these points in Region 4 is 61.75 dB μA, in which the difference between simulation and experiment results does not exceed 5 dB μA. The difference is also within 10 dB μA for other frequency points, which demonstrates the feasibility of the equivalent dipole model.

### Model error analysis

Observed from Fig. 14 that the radiation level is evenly distributed within the entire frequency range (around 32 dB μA), despite its maximum of 63 dB μA at 80 MHz. The radiation data obtained from near field measurement indicates that the main frequency is under 200 MHz and the radiation mainly originates from high-frequency devices like DSP. In order to compensate the frequency characteristics of the near-field probe and the connecting cable employed, the radiation voltage level needs to be transformed into radiation electric field. Hence, the following equation holds:

27 $$ E_{radiation} = U_{radiation} + R_{probe} + L_{waste} $$

where *E*_*radiation*_ stands for radiation electric field, *U*_*radiation*_ for radiation voltage displayed in the spectrum analyzer, *R*_*probe*_ for antenna factor of the probe, and *L*_*waste*_ for the cable loss. All of them are in logarithmic form.

In the experiment, near field probe RF-R50-1 by *Longer* is implemented with an antenna factor of 20 dB and cable loss of 3 dB. Therefore, for the radiation voltage level of 50 dB μA measured by the spectrum analyzer, the actual radiation level should be calculated as 50 dB μV + 20 dB + 3 dB = 73 dB μV/m. The model can be further verified by converting all the radiation voltages into the radiation electric field.

Arrange the radiation data in Fig. 14 by converting all radiation voltages to electric field. Take DSP as an example, the predicted radiation level reaches peak value of 82 dB μA/m with an average level of 68.2 dB μA/m from Fig. 14, while the measured level reach the peak of 84.75 dB μA/m with an average of 59.7 dB μA/m.

The radiation comparison implies that the peak difference and average difference between the theoretically predicted and measured vales do not exceed 6 dB. Since the uncertainty analysis yields an uncertainty limit of 6 dB, the model should be within engineering accuracy. The distinction between the predicted and measured values are within 10 dB for the entire frequency range, except for peak and average values, and the radiation frequency spectrum is essentially the same, which again verifies the effectiveness and accuracy of the proposed model.

## Conclusion

The feasibility of the presented modeling method of EMI of PCB has been verified by the good agreement of simulation and experimental results. The modeling method proposed in this paper successfully avoids the complicated settings for the boundary conditions, which is necessary when modeling EMI of PCB with traditional method. Although this method is partially based on experimental data, limited points are needed to predict the radiation level for the whole frequency range. The accuracy of this method is related to two factors: the number of dipoles and the number of measured points. There is no doubt that accuracy increases with available data, which, as a results, increase its complexity and costs. Therefore, necessary trade-off is expected in real applications.
